# Socio-Demographic Variables and Successful Aging of the Angolan Elderly

**DOI:** 10.1155/2016/5306756

**Published:** 2016-03-21

**Authors:** Laura Galiana, Melchor Gutiérrez, Patricia Sancho, Elizabeth-Hama Francisco, José M. Tomás

**Affiliations:** ^1^Department of Methodology for the Behavioural Sciences, University of Valencia, Blasco Ibañez 21, 46410 Valencia, Spain; ^2^Department of Developmental and Educational Psychology, University of Valencia, Blasco Ibañez 21, 46410 Valencia, Spain; ^3^Department of Psychology and Sociology, University of Zaragoza, FCSH Ciudad Escolar s/n, 44003 Teruel, Spain; ^4^Department of Educational Sciences, Higher Institute of the Education Sciences, University of Agostinho Neto, Luanda, Angola

## Abstract

The proportion of elderly people is growing faster than any other age group. Amongst them, the group of oldest old is indeed the segment of the elderly population with the fastest growth rate. The increase in the proportion of elderly in the Angolan population makes research on this area badly needed. Within the theoretical framework of successful aging, the study aims to test for sociodemographic group differences in perceived health, life satisfaction, and social relations in Angolan elderly. The dependent variables are three of the components of what has been called successful aging. Data came from a cross-sectional survey of elderly people living in Luanda. 1003 Angolan elderly were surveyed on sociodemographic information, perceived health, life satisfaction, and social support. MANOVAs were calculated to test for mean differences in the dependent variables. Results permit to conclude that the factors associated with the largest differences on the Angolan elderly's quality of life and social relations were age (becoming oldest old) and institutionalization. The interactions of several factors with age pointed out that the oldest old were clearly a group in which the decreased quality of life due to becoming oldest old could not be compensated by other factors, as it was the case in the group of young old.

## 1. Introduction

Aging is a global issue. In almost all countries, the proportion of elderly people is growing faster than any other age group [1]. Moreover, the oldest among the elderly portion of the population, here defined as those 80 years or older, is growing faster than its younger segment. Indeed, the average annual growth rate of people aged 80 years or over is twice as high as the growth rate of the population over 60 years of age [2]. Population aging is mainly a medical success but it is also a political, economic, and social challenge [3], and this may be especially true for developing countries that must cope with an aging population while still fighting against infectious disease control, malnutrition, and infant mortality [4].

The southern African region has the highest percentage of elderly people across the continent [5], and Angola is one of the countries in the area. With approximately 13 million inhabitants, 2.7 percent of Angola's population is over 65 years. In this fast aging world, older people will increasingly play a critical role in society and its development [6]. And, indeed, elderly Africans will “continue to make critical contributions to the welfare of their families, communities, and societies” [7, page 5]. But such contributions to development can only be ensured if elderly people enjoy adequate levels of health and life quality. However, research on the aging processes in Africa, and by extension in Angola, is very limited, and several authors have stressed the need for research in this context [4, 7, 8]. Therefore, studies on the associations of sociodemographic conditions on quality of life and social support should be encouraged.

The present study explores the associations of age, gender, marital status, educational level, and institutionalization, with quality of life and social support, and follows a great deal of research devoted to understand the elderly and, in particular, oldest old quality of life [9–12]. This literature has focused on both subjective indicators of life satisfaction and wellbeing [13] and health and functioning [14]. One of the main theoretical frameworks to understand wellbeing and its antecedents in the old age is that of successful aging [15]. Successful aging has been characterized as maintaining physical health, sustaining good physical and cognitive function, and having active engagement with other people and productive activities [16]. Additionally, wellbeing has been considered a potential indicator of psychological adaptation and successful aging [17, 18]. These theoretical contributions are also in line with the WHO definition of health as a state of complete physical, mental, and social wellbeing. All these theoretical contributions point the need of considering the potential effects of sociodemographic factors on health, wellbeing (life satisfaction), and social functioning.

Both health and life satisfaction, as indicators of wellbeing, have been related to a number of sociodemographic variables [19–23]. Age has been related to life satisfaction and most studies found a slight decrease in elderly's life satisfaction [24–26]. However, this negative relationship has not always been statistically significant [27].

Finally, there is a large amount of scientific evidence on the positive and large relationship between social support and social networks with life satisfaction. Given that active engagement with others is a component of successful aging [16, 28] and a supportive social network is important to keep it, the evidence on social relationships and networks is of relevance in this context. A wealth of studies in the elderly population has found that the quality and quantity of people's social network is related to life satisfaction in older adults [29–31]. Meta-analyses on life satisfaction have shown that social network and/or loneliness are consistently related to satisfaction with life [32, 33]. Therefore, the consideration of social support and social relationships as a dependent variable within the study seems adequate, given the potential effects of sociodemographic variables on this well-known determinant and/or dimension of quality of life and successful aging. Gender differences have sometimes aroused in the quantity of social networks. Apparently, women have larger networks than men [34]. Antonucci et al. [35] also found some differences between men and women in quantitative aspects of social networks, but not in qualitative ones. However, Meléndez-Moral et al. [36], in a study in Spanish population, found differences in the main role giving support to men (wife) compared to women (children). Nevertheless, several studies have also found gender differences in the quality of the social networks [37, 38]. A study by Cava and Musitu [39] found more formal and informal social support in the institutionalized elderly. However, this result could be dependent on living alone or not, as other studies have found [40].

Therefore, studies of the Angolan elderly population should be encouraged and welcomed, because of both the growing number of elderly people in this country and the lack of scientific research in this area in Angola and, by extension, in Africa. The aim of this study is to test for multivariate associations of several sociodemographic factors on perceived health, life satisfaction, and social relations in Angolan elderly. In particular the sociodemographic factors and related hypotheses are presented in the following.

- *Hypothesis One: Age.* Two groups have been considered, young old and oldest old (>79 years). Worse quality of life, that is, worse perceived health, life satisfaction, and social support, are hypothesized among the oldest old, as transition from the third to the fourth age appears to be a great psychological challenge. This is one of the main or primary factors in this research since it is also hypothesized that it may interact with other factors as, following the argument of Smith et al. [22], very old age may eventually be an overwhelming factor that dampens or limits the capacity to experience positive emotions.
- *Hypothesis Two: Gender*. Women will have worse perceived health and better social relations than men.
- *Hypothesis Three: Marital Status*. Widows and widowers are hypothesized to have lower average levels of life quality compared to married elderly.
- *Hypothesis Four: Education.* Educational level is hypothesized to improve quality of life, including a better perceived health, life satisfaction, and social relations.
- *Hypothesis Five: Living Conditions.* Institutionalization is hypothesized to produce lower perceived health and life satisfaction and higher levels of social relations.

## 2. Method

### 2.1. Design, Participants, and Procedure

The research design is a cross-sectional survey. The sampling procedure was nonprobabilistic, since neither a census of elderly living in Luanda nor a complete list of nursing homes and day-care centres was available. The 1003 participants were sampled from nursing homes dependent on Angola's Government, day-care centres depending on religious institutions, and NGO assisting elderly living alone, in Luanda (Angola). With the previous agreement of the Ministry of Social Welfare (Ministério de Reinserção Social), several old people's homes from the city of Luanda were asked to participate in the research, as well as day-care centres depending on religious institutions and NGO assisting elderly living alone. Some surveys were completed in nursing homes while others were completed at the people's homes. When elderly's age and/or cultural level made self-completion difficult, trained interviewers were used. A typical interview session was used to last around thirty minutes.

In the sample, 73.5 percent were young old. 65.4 percent were women. With respect to marital status, most of the participants were widows or widowers (62.4 percent), 25.1 percent were single or divorced, and the remaining 12.5 percent were married at the time of the study. 43.9 percent had low level of studies, medium educational level was 40.6 percent of the sample, and only 15.6 percent had high educational level. 76.3 percent were living in nursing homes.

### 2.2. Instruments

The scales in the study were back-translated into Portuguese, with the exception of the satisfaction with life scale; in this case, the author's translation was used [41]. The measures were as follows.

(a) *Sociodemographic Variables*. Gender; marital status (widowed, single/divorced, and married); educational level (low, medium, and high); institutionalization (institutionalized* versus* community dwelling); and age (60 to 79 years old* versus* 80 years old or over): the age categorization into two subgroups responds to a qualitative interest to study the differences between the oldest old group and the so-called young old. Several cut-off criteria have been used to distinguish the group of oldest old. 85-year-old or over criterion, for example, is used by the WHO. However, 80-year-old or over criterion is also frequently used, for example, in the Survey of Health, Ageing, and Retirement in Europe [42]. Given that Angola has a life expectancy of 51 years for men and 53 years for women, much lower than life expectancy of Western countries, it seems logical to use a relatively lower cut-off. Additionally, using this cut-off point resulted in a larger subsample of oldest old.
(b) *Satisfaction with Life Scale [43, 44]*. The satisfaction with life scale is composed of five items assessing global life satisfaction, using a Likert-type response format. Items scored from one* (totally disagree)* to seven* (totally agree)*. The alpha was .92. An example item is “*In most ways my life is close to my ideal.*”
(c) *Perceived Health Scale [45]*. The scale assesses elderly's perception of health with five items scoring from one to three. It has also shown high internal consistency, *α* = .77. An example item is* “In the last twelve months your health has been…,”* from* bad* (one) to* good* (three).
(d) *The Emotional Support Scale, Formed by Four Items That Asked Participants about the Emotional Support Received From Their Friends [46]*. Each item scores on a four-point Likert scale, ranging from one* (not at all)* to four* (a lot)*. Internal consistency was .84. An example item is* “How much do your friends really care about you?”*
(e) A two-item indicator of perceived adequacy of social relations was adapted from the Aging Perception Scale [47]. The two indicators were* “I have a good relationship with my closest relatives”* and* “I think the relationship with my friends is good”* and both were measured with a seven-point Likert scale from one* (totally disagree)* to seven* (totally agree)*. The alpha was .72.

Descriptive statistics for the variables under study can be consulted in Table 1.

### 2.3. Analyses

Multivariate analyses of variance (MANOVA) were used to test the main and interaction effects of sociodemographic categorical variables on several dependent variables: perceived health, life satisfaction, social relations, and emotional support from friends. MANOVA tests the differences in the centroid (vector) of means of the multiple dependents, for various categories of the independents. Pillai's criterion test was used because it is the most robust to violations of the underlying assumptions [48]. When the overall *F*-test showed that the centroid (vector) of means of the dependent variables was not the same for all the groups, post hoc univariate *F*-tests of group differences were used to determine which group means differ significantly from others. Subsequently, pairwise multiple comparison tests were used to further investigate differences among groups. Effect sizes (partial eta-squares) were estimated. Cohen's guidelines to interpret the magnitude of those effects were employed: .02, .13, and .26 for small, medium, and large effects, respectively [49]. All statistical analyses were performed in SPSS 20. Given the available sample size and the number of categories in the independent variables, it was not possible to estimate a single 2 (age group) × 2 (gender) × 3 (marital status) × 2 (institutionalization) × 3 (educational level) MANOVA. Therefore, as age group was considered the main independent variable, several MANOVAs were calculated: (a) 2 (age group) × 2 (gender); (b) 2 (age group) × 3 (marital status); (c) 2 (age group) × 2 (institutionalization); (d) 2 (age group) × 3 (educational level).

## 3. Results

A first 2 (age) × 2 (gender) between-subjects MANOVA was performed on four dependent variables: satisfaction with life, perceived health, social relations, and emotional support from friends. Correlations among these dependent variables can be consulted in Table 2. Independent variables were age (79 years or less and 80 years or older) and gender (women and men). Using of Pillai's criterion, the combined dependent variables were significantly affected by both age (*F*4, 996 = 115.33, *p* < .001) and gender (*F*4, 996 = 5.87, *p* < .001), as well as their interaction (*F*4, 996 = 9.34, *p* < .001). Results reflected a large association between age and the dependent variables, with partial *η* ^2^ = .32. The interaction explained a modest amount of variance, as reflected in the partial *η* ^2^ = .036. The association was even less substantial between gender and the dependent variables (partial *η* ^2^ = .023).

To investigate the impact of main and interaction effects on the individual dependent variables, analyses of variance (ANOVA) were performed. The results of these additional analyses are shown in Table 3. Means and standard deviations for the main effects are presented in Table 4. Age had a significant effect on the four dependent variables (*p* < .05), with averages higher in young old in all variables. With respect to the main effects of gender, these were quite small and were statistically significant in perceived health, with men informing a slightly higher mean than women, and emotional support from friends, with women informing a slightly higher mean than men. The interactions of gender and age with satisfaction with life and emotional support were statistically significant. The decrease in both life satisfaction and emotional support associated with age was steeper for men than it was for women.

A second 2 (age) × 3 (marital status) between-subjects MANOVA was performed on the same dependent variables. The main effects regarding age are not repeated since they are redundant with the first MANOVA. With the use of Pillai's criterion, the combined dependent variables were significantly affected by marital status (*F*8, 1990 = 5.79, *p* < .001) and by their interaction (*F*8, 1990 = 3.87, *p* < .001). The results reflected a lower association between marital status and the combined dependent variables, with partial *η* ^2^ = .02. The interaction effect was even less substantial (partial *η* ^2^ = .01).

To investigate the impact of main and interaction effects on the individual dependent variables, ANOVAs were performed (Table 3). With respect to the main effects of marital status, these were statistically significant in perceived health and social relations. Married people reported higher perceived health than the rest, and single/divorced and married informed better social relationships than widows and widowers. There were significant interactions of marital status and age on satisfaction with life and social relations. The decrease in both life satisfaction and social relations associated with being among the oldest old was steeper for married elderly people than it was for the other groups (single/divorced and widows and widowers). A third 2 (age) × 2 (institutionalization) between-subjects MANOVA was performed on the same dependent variables. As indicated above, the main effects of age group are not presented for the sake of clarity. With the use of Pillai's criterion, the combined dependent variables were significantly affected by institutionalization (*F*4, 996 = 12.90, *p* < .001) and by the age-institutionalization interaction (*F*4, 996 = 9.36, *p* < .001). The results reflected a lower association between institutionalization and the combined dependent variables, with partial *η* ^2^ = .05. The effect size for the interaction was also quite low (partial *η* ^2^ = .04).

Four ANOVAs were performed to study the impact of main and interaction effects on the individual dependent variables (Table 3). Institutionalization had a significant main effect in satisfaction with life, perceived health, and social relations (*p* < .05), with higher means for the noninstitutionalized elderly (see Table 4). The age *∗* institutionalization interactions were statistically significant on life satisfaction, social relations, and emotional support. In all cases, there was a significant difference that favored the noninstitutionalized elderly in the group of the young old. This difference disappeared in the group of the oldest old.

A fourth, and last, 2 (age) × 3 (educational level) between-subjects multivariate analysis of variance was performed on the same dependent variables. As in previous analyses, main effect for age group is not presented. With the use of Pillai's criterion, the combined dependent variables were significantly affected by educational level (*F*8, 1990 = 12.46, *p* < .001) and by their interaction (*F*8, 1990 = 10.64, *p* < .001). The results reflected a lower association between educational level and the combined dependent variables, with partial *η* ^2^ = .05. The interaction effect was even less substantial (partial *η* ^2^ = .04).

To investigate the impact of main and interaction effects on the individual dependent variables, univariate analyses of variance were performed. The results of these additional analyses are shown in Table 3. Educational level had a significant main effect in all four dependent variables (*p* < .05). The overall pattern was that means increased as education level increased (see Table 4). There were significant interaction effects of educational level and age on satisfaction with life, perceived health, and social relations. The interaction was due to a steeper loss of satisfaction with life and social relationship in the group of high educational level. That is, the decrease in life satisfaction and social relations produced by aging was larger for the group of high educational level. On the contrary, the decrease in perceived health was less important for the group of high educational level than it was for the other educational levels.

## 4. Discussion

According to data from the United Nations [50], the aging process of the world population is more acute for the African subregion. The results of this study highlight the associations of sociodemographic factors with the elderly's quality of life in this African context, and they should be interpreted in light of the successful aging theoretical framework. The importance of current results is magnified when the African cultural, and Angolan sociohistorical, contexts are considered. The elderly in the present sample have all lived through 40-year period of wars in Angola. Together with this period of war, Angola has had profound sociological and economical changes, among these, a rapid urbanization, which has been common across all Sub-Saharan countries [51] and is thought to have weakened the family ties that sustained the elderly in the past [4], because of a decrease in the number and proximity of members of the social networks, as well as a shift in the traditional cultural African view according to which the elderly will be looked after by their offspring [52]. Additionally, the Sub-Saharan region has suffered a high mortality rate of adults due to HIV/AIDS that has produced huge numbers of orphans that are usually cared for by grandparents [53]. In sum, the Sub-Saharan elderly are “characterized by growing inadequacies in customary family support systems, vulnerability to poverty, and exclusion from health services” [7, p.i]. Taking into account that people with different value systems and cultural backgrounds may perceive and understand successful aging in different ways, studies on other cultures different from occidental countries should be welcomed [54].

With respect to hypothesis one, a main result was that age and, specifically, being among the oldest old, had a large effect on the quality of life. The oldest old had worse perceived health, life satisfaction, social relations, and emotional support. This overall result was similar to the majority of evidence found in health literature [20, 22, 23]; it was also consistent with the slight decrease in life satisfaction found in several studies [24–26] and with the literature on social relations and networks and aging [55–57], although here the evidence was somewhat indirect. This evidence is in line with the study of Bowling and Browne [56], who noted that close relationships are essential for effective support and that this close support is usually given by age-mates (see also [55]). As a consequence, the oldest old are at higher risk of losing this support. Greenblatt et al. [57] confirmed that spouses and members of the immediate family provide the most support in times of crisis. Informal social participation, such as contact with family and friends, actually increases in response to widowhood at older ages, while formal participation remains constant [58].

We agree with Smith et al. [22] that the experience of the positive side of life was especially compromised in the group of the oldest old.

With respect to gender, data partially support hypothesis two, since there were some significant differences (on perceived health and emotional support), as hypothesized, but all of them were of a rather small magnitude (accounting for around one percent of the variance in the dependent variables). These differences are in line with those found by Antonucci et al. [35] and Nygren et al. [59] on health measures. Literature on gender differences on perceived health has generally shown less perceived health for women, a result also found in elderly women [35, 59–61]. Results are also consistent with the result found by Troll [34], according to whom women had larger social networks. Gender differences, however, were not found in elderly's life satisfaction, which is in line with some of previous literature [27], but not with others [25]. Two age and gender interactions were also found to be significant, but again they explained very little variance. These interaction effects on life satisfaction and emotional support both indicated that men had a steeper decrease compared to women.

With respect to differences due to marital status (hypothesis three), evidence also partially supported the hypothesized effects, but again the amount of variance in the dependent variables explained was quite low. However, widows and widowers' perceived health and social relations were more compromised than the ones married or single/divorced. This result supports several previous studies, in which less perceived health and more depression are found among widows and widowers [62]. Carr et al. [63] explored differences among widows and married controls and found more anxiety, depression, and yearning in widows and widowers. In this line, Greenblatt et al. [57] found that spouses are one of the most important figures giving support and that figure is lost when the spouse dies. Thus, marital status, and specially widowhood, seems to be associated with objective and subjective measures of health, also in Angolan elderly. As regards life satisfaction, no effect of marital status was found. This contradicts previous studies [16, 25, 27, 64], such as the study carried by Berg et al. [24], in which authors found a significant linear decline in the oldest old life satisfaction as age increased, and this linear decrease was affected by the loss of a spouse, in particular in men. There were also two significant interactions of age and marital status on life satisfaction and social relations, but again these accounted for very little variance. The interaction effects, nevertheless, were clearly interpretable: the advantage that married young old had in life satisfaction and social relations disappeared in the group of the oldest old.

Educational level (hypothesis four) had a main effect quite limited in its strength, but consistent with the hypothesis and with the literature on the positive association of educational level and income on quality of life (it could be argued that the coarse measure of educational level in this study is a proxy) found in several studies [25, 26, 65, 66]. That is, a positive but low association was found between educational level and life satisfaction and wellbeing, such as in previous literature [25, 26]. Finally, there is evidence on the positive effects of socioeconomic status and educational level as one of its proxies, on health outcomes [65–67]. Oliver et al. [26] found a link between educational level and the ability to perform daily life activities. In our results, several interactions of educational level and age were found although they were of small magnitude. The interactions showed that the decrease in life satisfaction and social networks produced by aging (becoming oldest old) was steeper for highly educated elderly. Interestingly, the contrary happened for perceived health: highly educated elderly perceived better health, a pattern that could be associated with potential wealth being related to higher education in Angola.

Finally, and contrary to previous results [39], institutionalization had relatively larger effect sizes, especially on perceived health and social relations, and the results were quite clear: institutionalized elderly had worse conditions than the ones living in their homes. This result is consistent with some of the available literature on health related to the institutionalized elderly's quality of life, which pointed out that being institutionalized is negatively associated with health [40, 68, 69]. Naleppa [68] described several studies referring to poor health conditions as main causes of elderly's institutionalization. Other studies have been less conclusive, either finding no differences for health conditions [39] or exclusively on activity levels [70]. In this study, however, age moderated these relationships. The significant interaction effects suggested that the noninstitutionalized elderly's advantage over the ones in nursing homes disappeared when the group studied was the oldest old.

Overall, the results permit us to conclude that the factors with largest effect sizes on quality of life and social relations of the Angolan elderly were age (becoming oldest old) and institutionalization, and in general the interactions of several factors with age pointed out that the oldest old were clearly a group in which the decreased quality of life cannot be compensated by other factors, as it is the case for the youngest old. This result is in line with the argument by Smith et al. [22] that the oldest old may have reached the limit of adaptation to declining health. And this, obviously, includes the possibilities for social relations. Social participation and social contacts, for example, are prime sources of positive affect.

This study reported has a number of strengths and limitations. Among the strengths, the research is developed in an African country, Angola. Although the aging process in Africa has been slower, its elderly population is estimated to reach 6 percent in 2025 and 11 percent in 2050. Among the outcomes of the Oxford Conference on* Research on Ageing, Health, and Poverty in Africa*, there is recognition of the lack of evidence and knowledge in this arena and the “vital need for enhanced research on aging in Africa” [7, page 2]. In the same vein, Stuart-Hamilton as Editor of a recent manual of Gerontology encourages authors to “try where possible to include a significant cross-cultural element in their writing… because research has been almost exclusively concentrated in a limited number of Western industrialized cultures” [71, page 432]. Another strength of this study is the stability of the effects of the sociodemographic factors on the dependent variables across cultures and continents. The results found in this study were mainly according to hypotheses found in quite different cultural contexts (mainly Western societies). However, this study also has limitations, among them, the cross-sectional nature of the research design; this makes it extremely difficult to establish causal links. Other shortcomings are the nonprobabilistic nature of the sample or the absence of information on elderly's medical condition. Further studies including information on comorbidities, drugs assumption, disability, functional status, and data from rural areas are needed. This would enrich the knowledge about the process of aging, together with a deeper understanding of the effects of the urbanization process in Angola and Sub-Saharan Africa [51]. Random sampling and longitudinal monitoring would allow further insights into the causal processes underlying the associations found.

## Figures and Tables

**Table 1:** Descriptive statistics for the variables under study.

	Min	Max	Mean	SD	Asim	Asim error	Kurt	Standard error
Perceived health	1.00	2.80	1.88	0.45	−0.388	.077	−0.599	.154
Life satisfaction	1.00	6.80	3.33	1.55	0.305	.077	−0.951	.154
Social relations	1.00	7.00	3.55	1.58	0.489	.077	−0.612	.154
Emotional support from friends	1.00	3.25	1.85	0.57	0.335	.077	−0.267	.154

Notes: Min = minimum score; Max = maximum score; SD = standard deviation; Asim = asymmetry; Asim error = asymmetry error; Kurt = kurtosis.

**Table 2:** Correlations among dependent variables.

	Perceived health	Life satisfaction	Social relations	Emotional support from friends
Perceived health	1			
Life satisfaction	.326^*∗∗*^	1		
Social relations	.294^*∗∗*^	.696^*∗∗*^	1	
Emotional support from friends	.288^*∗∗*^	.416^*∗∗*^	.382^*∗∗*^	1

Notes: ^*∗∗*^ *p* < .01.

**Table 3:** Follow-up ANOVAs.

Sources of variation	Dependent variables	df_effect_	df_error_	*F*	*p*	Partial η^2^
Gender	Satisfaction with life	1	999	1.778	.183	.002
	*Perceived health*	*1*	*999*	*5.751*	*.017*	*.006*
	Social relations	1	999	1.551	.213	.002
	*Emotional support from friends*	*1*	*999*	*5.083*	*.024*	*.005*
Age	*Satisfaction with life*	*1*	*999*	*339.448*	*.000*	*.254*
	*Perceived health*	*1*	*999*	*171.733*	*.000*	*.147*
	*Social relations*	*1*	*999*	*165.187*	*.000*	*.142*
	*Emotional support from friends*	*1*	*999*	*28.566*	*.000*	*.028*
Gender *∗* age	*Satisfaction with life*	*1*	*999*	*21.316*	*.000*	*.021*
	Perceived health	1	999	2.039	.154	.002
	Social relations	1	999	0.429	.513	.000
	*Emotional support from friends*	*1*	*999*	*7.025*	*.008*	*.007*

Marital status	Satisfaction with life	2	997	1.268	.282	.003
	*Perceived health*	*2*	*997*	*7.639*	*.001*	*.015*
	*Social relations*	*2*	*997*	*6.871*	*.001*	*.014*
	Emotional support from friends	2	997	0.005	.995	.000
Age	*Satisfaction with life*	*1*	*997*	*112.388*	*.000*	*.101*
	*Perceived health*	*1*	*997*	*51.234*	*.000*	*.049*
	*Social relations*	*1*	*997*	*32.807*	*.000*	*.032*
	*Emotional support from friends*	*1*	*997*	*8.927*	*.003*	*.009*
Marital status *∗* age	*Satisfaction with life*	*2*	*997*	*6.872*	*.001*	*.014*
	Perceived health	2	997	2.062	.128	.004
	*Social relations*	*2*	*997*	*4.722*	*.009*	*.009*
	Emotional support from friends	2	997	0.950	.387	.002

Institutionalization	*Satisfaction with life*	*1*	*999*	*11.929*	*.001*	*.012*
	*Perceived health*	*1*	*999*	*21.463*	*.000*	*.021*
	*Social relations*	*1*	*999*	*30.332*	*.000*	*.029*
	Emotional support from friends	1	999	0.410	.522	.000
Age	*Satisfaction with life*	*1*	*999*	*191.634*	*.000*	*.161*
	*Perceived health*	*1*	*999*	*38.947*	*.000*	*.038*
	*Social relations*	*1*	*999*	*73.426*	*.000*	*.068*
	*Emotional support from friends*	*1*	*999*	*18.239*	*.000*	*.018*
Institutionalization *∗* age	*Satisfaction with life*	*1*	*999*	*32.555*	*.000*	*.032*
	Perceived health	1	999	1.659	.198	.002
	*Social relations*	*1*	*999*	*7.556*	*.006*	*.008*
	*Emotional support from friends*	*1*	*999*	*6.760*	*.009*	*.007*

Educational level	*Satisfaction with life*	*2*	*997*	*6.060*	*.002*	*.012*
	*Perceived health*	*2*	*997*	*31.428*	*.000*	*.059*
	*Social relations*	*2*	*997*	*22.638*	*.000*	*.043*
	*Emotional support from friends*	*2*	*997*	*6.763*	*.001*	*.013*
Age	*Satisfaction with life*	*1*	*997*	*368.370*	*.000*	*.270*
	*Perceived health*	*1*	*997*	*140.297*	*.000*	*.123*
	*Social relations*	*1*	*997*	*192.351*	*.000*	*.162*
	*Emotional support from friends*	*1*	*997*	*25.306*	*.000*	*.025*
Educational level *∗* age	*Satisfaction with life*	*2*	*997*	*20.621*	*.000*	*.040*
	*Perceived health*	*2*	*997*	*10.918*	*.000*	*.021*
	*Social relations*	*2*	*997*	*8.032*	*.000*	*.016*
	Emotional support from friends	2	997	1.669	.189	.003

Notes: statistically significant relationships appear in italics; df_effect_ = degrees of freedom of the effect; df_error_ = degrees of freedom of the error.

**Table 4:** Means and standard errors of the dependent variables on each group of the sociodemographic factors.

Factors	Perceived health		Life satisfaction		Social relations		Support	
	Mean	SE	Mean	SE	Mean	SE	Mean	SE
*Age*								
79 or less	2.003	.016	3.847	.053	3.950	.057	1.904	.022
80 or older	1.593	.027	1.995	.086	2.544	.093	1.680	.036
*Gender*								
Men	1.836	.025	2.854	.080	3.315	.088	1.745	.034
Women	1.761	.019	2.988	.060	3.179	.066	1.839	.025
*Marital status*								
Single/divorced	1.619	.049	2.693	.154	3.671	.170	1.789	.067
Widow/widowed	1.792	.017	2.847	.053	3.068	.059	1.795	.023
Married	1.956	.086	3.184	.271	3.610	.298	1.791	.118
*Institutionalization*								
No	1.992	.049	3.273	.148	3.941	.163	1.820	.067
Yes	1.751	.016	2.734	.048	3.000	.053	1.775	.022
*Educational level*								
Low	1.674	.022	2.767	.070	2.869	.076	1.723	.030
Medium	1.816	.024	3.121	.078	3.505	.084	1.875	.033
High	1.987	.034	3.033	.111	3.645	.120	1.864	.047

Note: SE = standard error.
